# Genital pseudomyogenic hemangioendothelioma: case report and literature review^؆^

**DOI:** 10.1016/j.abd.2026.501303

**Published:** 2026-03-23

**Authors:** Javier de la Iglesia-Martin, Irene Fuertes de Vega, Agustí Toll-Abello, Alba Catala-Gonzalo, Raquel Albero-Gonzalez

**Affiliations:** a Department of Dermatology, Hospital Clínic Barcelona, University of Barcelona, Barcelona, Spain; b Department of Pathology, Hospital Clínic Barcelona, University of Barcelona, Barcelona, Spain

*Dear Editor,*

Pseudomyogenic Hemangioendothelioma (PHE) is a rare neoplasm that typically presents as nodules on the extremities. Genital involvement is rare. We present the case of a young man with a localized PHE on the penis.

A 23-year-old male presented for consultation regarding sexually transmitted diseases. He reported an asymptomatic ulcer on his penis with a 2-week duration. He had no history of Sexually Transmitted Infections (STIs). Over the previous three months, he had engaged in sexual activity with two female partners.

On physical examination, a round ulcer with raised edges at the inner foreskin was evident (Fig. 1). No urethral discharge or lymphadenopathy was observed. An empirical dose of benzathine penicillin G (2.4 million units intramuscularly) was administered. An STIs screening was performed.

**Fig. 1:**
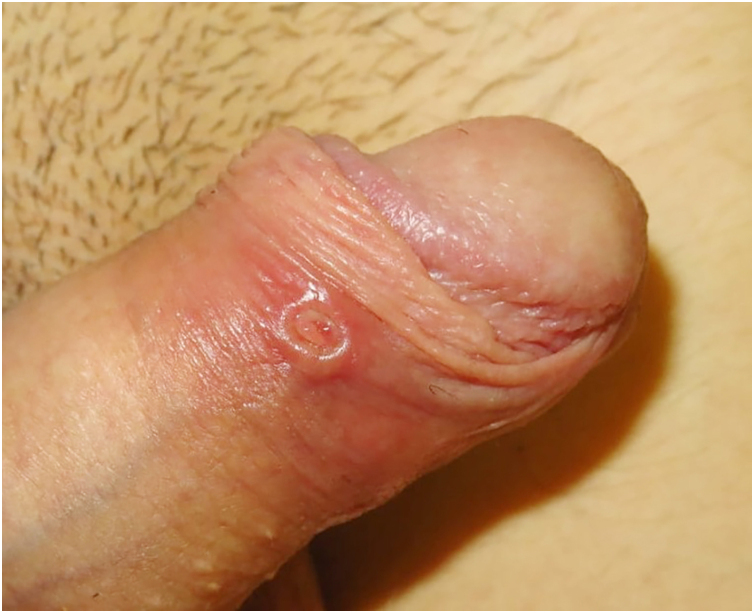
Circular ulcer with raised edge at the inner foreskin.

One week later, the patient reported no improvement. PCR testing of the ulcer was negative for *Treponema pallidum*, *Haemophilus ducreyi*, *Chlamydia trachomatis* serovars L1, L2, and L3, as well as herpes simplex virus types 1 and 2. Serologic testing for syphilis, hepatitis, and HIV was negative.

A biopsy revealed a multinodular, poorly defined dermal lesion. It consisted of epithelioid and spindle-shaped cells with abundant eosinophilic cytoplasm, moderate nuclear atypia with prominent nucleoli, and isolated mitotic figures. There was a prominent infiltrate of neutrophils, numerous vascular structures with interspersed prominent endothelial (tack) cells, and areas of central fibrosis. The adjacent epidermis exhibited focal parakeratosis, mild spongiosis, and associated focal lymphocytic exocytosis (Fig. 2). PAS, Grocott, Gram, and Ziehl-Neelsen (BK) stains showed no microorganisms.

**Fig. 2:**
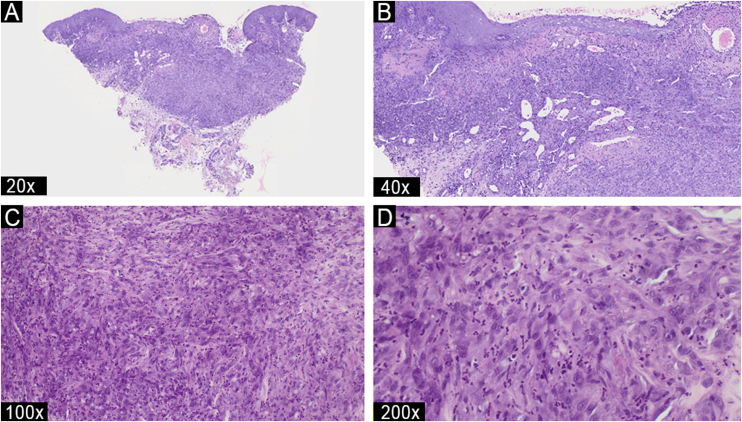
Light microscopy – (A) Hematoxylin & eosin (Hematoxylin & eosin, ×20) image of the ulcerated dermal lesion with multinodular architecture and poorly defined borders. (B) (Hematoxylin & eosin, ×40) Prominent polymorphonuclear infiltrate; vascular structures with interspersed prominent endothelial (tack) cells, and areas of central fibrosis. (C‒D) (Hematoxylin & eosin, ×100, ×200) Higher power section of atypical spindle and epithelioid cells, some of them with dense eosinophilic cytoplasm and eccentric nuclei, resembling rhabdomyoblasts.

Immunohistochemistry was negative for herpes viruses 1, 2, and 8, cytomegalovirus, and Treponema. CKAE1/AE3 showed weak, diffuse positivity, while SOX10 and CD45 were negative. Moderate, diffuse positivity for ERG and CD31 and strong, diffuse positivity for FOSB (Fig. 3), with weak positivity for smooth muscle actin and retained SMARCA4 expression. Stains for p40, D2-40, CD34, desmin, and H-caldesmon were negative. CD3, CD20, and CD30 highlighted sparse accompanying inflammatory cells (Table 1). Next-generation sequencing revealed an ACTB–FOSB gene fusion.

**Fig. 3:**
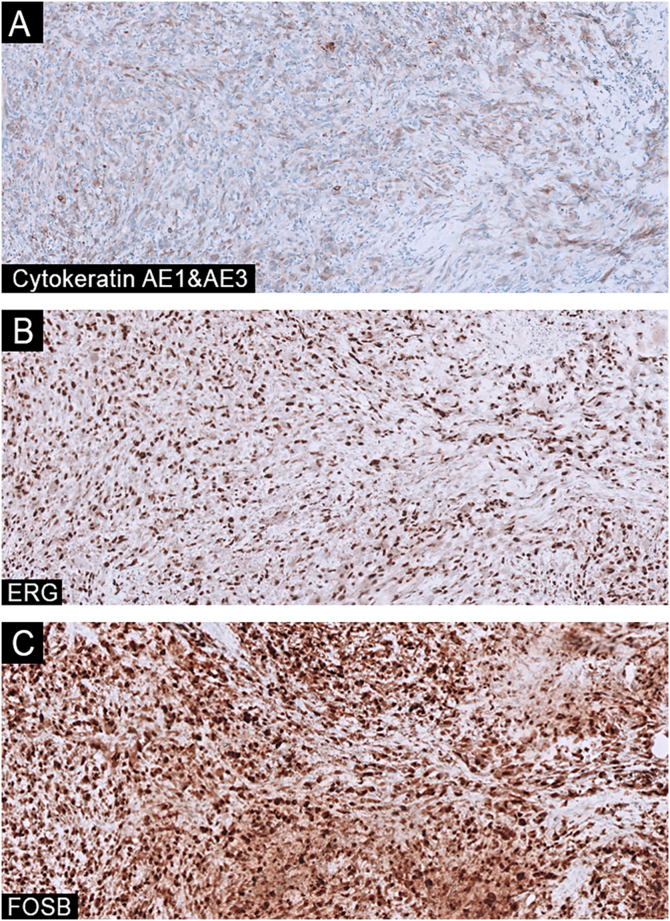
Imunnohistochemistry -The tumor cells showed weak and diffuse positivity for Cytokeratin AE1/AE3 (A, ×100), moderate and diffuse positivity for ERG (B, ×100), and strong and diffuse positivity for FOSB (C, ×100).

**Table 1:** Antibodies, clones, results, presentations, and producers.

Antibody	Clone	Result	Presentation	Producer
CKAE1/AE3	AE1+AE3	Weak, diffuse positivity	Prediluted	Diagnostic BioSystems
SOX10	SP267	Negative	Prediluted	Ventana
CD45	2B11&PD7/26	Negative	Prediluted	Ventana
ERG	EPR3864	Moderate, diffuse positivity	Prediluted	Ventana
CD31	JC70	Moderate, diffuse positivity	Prediluted	Ventana
FOSB	5G4	Strong, diffuse positivity	Concentrated (dilution: 1/50)	Cell Signaling
P40	BC28	Negative	Prediluted	Ventana
D240	D240	Negative	Prediluted	Ventana
CD34	QBEnd/10	Negative	Prediluted	Ventana
Desmin	DE-R-11	Negative	Prediluted	Ventana
H-Caldesmon	hHCD	Negative	Prediluted	Diagnostic BioSystems
SMARCA4	EPNCIR111A	Positive	Concentrated (dilution:1/100)	Abcam
INI-1	MRQ-27	Positive	Prediluted	Ventana
CD3	2GV6	Moderate, diffuse positivity	Prediluted	Ventana
CD20	L20	Moderate, diffuse positivity	Prediluted	Ventana
CD30	JCM182	Moderate, diffuse positivity	Concentrated (dilution: 1/15)	Leica

Diagnosis of PHE was reached. The lesion was excised with clear margins after two rounds of Mohs surgery.

PHE is a locally aggressive endothelial neoplasm^1^, ^2^ that predominantly presents as nodules on the lower extremities. Extension into the underlying bone is relatively frequent.

Genital involvement is rare. Eight cases have been documented (Table 2).^2^, ^3^, ^4^, ^5^, ^6^, ^7^ It usually presents as non-ulcerated, asymptomatic nodules. In males, the predominant location is the glans penis; in females, the labia majora.^3^ The ulcerated and non-nodular presentation is exceptional and can mimic more common entities such as squamous cell carcinoma, cutaneous sarcomas, STIs, or traumatic ulcers. This atypical clinical form can delay diagnosis or lead to initial diagnostic errors, given that the ulcerated appearance is not usually associated with low-grade vascular tumors.

**Table 2:** Genital pseudomyogenic hemangioendothelioma reported cases.

Authors, year	Age	Sex	Onset	Symptoms	Signs	Location	Pathology	Treatment	Recurrence	Follow-up
Zhou et al (2024)^3^	36	F	NS	NS	Violet nodule	Labia majora	Epithelioid cells arranged in cords with intracytoplasmatic vacuoles	WLE and reexcision (recurrence)	Yes	ANED
							Positive for CK, ERG, Fli-1, INI-1 and vimentin+			
	37	M	NS	NS	2 nodules	Glans	Spindle cells with bright eosinophilic cytoplasm	WLE	No	ANED
							Positive for CK(AE1/AE3), CD31, ERG, Fli-1, INI-1			
Song et al (2020)^4^	30	M	2m	Itchiness	Nodule	Glans	NS	No	No	AWD
Sun et al (2019)^5^	51	F	3m	Itchinesss, pain	2 nodules of 1cm	Labia majora	Fascicles of epithelioid cells with eosinophilic cytoplasm.	Marginal resection	No	3m ANED
							Positive for CK, ERG and Fli1+			
Wang XL et al (2019)^6^	26	F	12m	No	NS	Perineum	NS	WLE	No	17 m ANED
Ide et al (2015)^7^	43	M	6m	Pain	3 Nodules of a few mm	Penis	Rhabdomyoblast-like atypical mesenchymal cells proliferating and infiltrating in the dermis and subcutis. CK, CD31, ERG, Fli1, INI1 EMA, vimentin +	WLE	Yes	9m ANED
Hornick et al (2011)^2^	27	M	NS	NS	Nodules	Penis	NS	NS	Yes	ANED
	18	M	NS	NS	Nodule	Penis/ Scrotum	NS	WLE	NS	ANED
Current Report	23	M	0.5m	No	Ulcerated papule	Glans	Epithelioid and fusiform cells with eosinophilic cytoplasm, neutrophils, central fibrosis. CK, ERG, CD31, FOSB, SMA	Mohs (conventional)	No	ANED

M, Male; F, Female; m, Months; ANED, Alive with No Evidence of Disease; WLE, Wide Local Excision; NS, Not Said; cm, Centimetre; mm, Milimeters.

Histopathologically, genital PHE is similar to cases reported in other locations. At low magnification, the tumor appears as a poorly circumscribed dermal and hypodermal proliferation. At higher magnification, it shows numerous cells with abundant eosinophilic cytoplasm, reminiscent of rhabdomyoblasts. In some areas, vascular lumina are infiltrated by tumor cells. The infiltrative nature of the tumor may extend into muscle, fat, and occasionally bone, with sporadic intracytoplasmic vacuoles and rare lumina.^8^

Immunohistochemically, PHE shows dual expression of endothelial markers (ERG, FLI1) and cytokeratins (most commonly AE1/AE3), aiding in differentiation from other vascular and soft tissue tumors. Focal expression of smooth muscle actin is present in up to one-third of cases. Nuclear positivity for FOSB is a consistent and distinctive feature. Retained INI1 (SMARCB1) expression also helps distinguish PHE from morphologically similar entities, mainly conventional and proximal type epithelioid sarcoma.^9^

Molecularly, recurrent FOSB gene fusions are a hallmark of PHE, contributing to its pathogenesis and providing diagnostic and potentially therapeutic targets. Translocations involving chromosomes 7 and 19 are common and may play a role in tumor development.^9^

Surgery remains the mainstay of treatment.^10^ PHE may exhibit local aggressiveness and a high recurrence rate if not completely excised, but distant metastases are rare.

A high index of suspicion is essential in young patients presenting with persistent genital nodular lesions of several weeks’ or months’ duration, particularly when serologic testing for STIs is negative and empirical antimicrobial treatment is ineffective. In such cases, a biopsy should be performed to rule out neoplasms.

## ORCID IDs

Irene Fuertes de Vega: 0000-0002-9314-1843

Agustí Toll-Abello: 0000-0003-2656-0076

Alba Catala-Gonzalo: 0000-0002-5132-2814

Raquel Albero-Gonzalez: 0000-0001-9027-1426

## Authors' contributions

Javier de la Iglesia-Martin: Methodology; writing-original draft; writing-review and editing; image editing.

Irene Fuertes de Vega: Methodology; writing, review, and editing.

Agustí Toll-Abello: Data curation; review.

Alba Catala-Gonzalo: Methodology; writing, review, and editing.

Raquel Albero-Gonzalez: Methodology; writing-original draft; writing-review and editing; image acquisition.

## Financial support

None declared.

## Research data availability

Does not apply.

## Conflicts of interest

None declared.
